# Factors Influencing the Selection of Materials and Luting Agents for Single-Crown Restorations

**DOI:** 10.3390/dj13050207

**Published:** 2025-05-09

**Authors:** Ahmad Alsahli, Mirza Rustum Baig, Jagan Kumar Baskaradoss, Shoug Alsanea, Athoub AlMousawi

**Affiliations:** 1 Department of General Dental Practice, College of Dentistry, Kuwait University, P.O. Box 24923, Safat, Kuwait City 13110, Kuwait; 2 Discipline of Prosthodontics, Department of Restorative Sciences, College of Dentistry, Kuwait University, P.O. Box 24923, Safat, Kuwait City 13110, Kuwait; mirza.baig@ku.edu.kw; 3 Discipline of Dental Public Health, Department of Developmental and Preventive Sciences, College of Dentistry, Kuwait University, P.O. Box 24923, Safat, Kuwait City 13110, Kuwait; jagan.baskaradoss@ku.edu.kw; 4 Kuwait Institute for Medical Specializations (KIMS), P.O. Box 1793, Safat, Kuwait City 13018, Kuwait; s.alsanea96@gmail.com; 5 Ministry of Health, P.O. Box 5, Safat, Kuwait City 13001, Kuwait; dr.athoubb@gmail.com

**Keywords:** crown, ceramics, dental cements, dental materials, prosthodontics, luting agent, zirconia, lithium disilicate, dual cure, light cure

## Abstract

**Objective:** Selecting suitable materials and luting agents for single crowns is critical yet challenging, as dentists must consider different factors. This study aimed to assess dentists’ preferences for materials and luting agents under different clinical conditions and evaluate the nonclinical factors influencing their preferences. **Methods:** A paper-based survey supplemented with photographs illustrating anterior and posterior single-crown preparation designs was used, incorporating three clinical scenarios for each as examples. Participants provided demographic data and were asked to select their preferred material and luting agent for each scenario. Comparisons between the crown material/luting agent choices and dentist/practice characteristics were performed. Significant differences were determined using the chi-square test. **Results:** Overall, 262 (87.3%) dentists participated in this survey. The top-selected material for anterior preparation designs was lithium disilicate; monolithic zirconia was the most selected for posterior preparation designs. Dual-cure resin was the most selected luting agent for all anterior and posterior clinical scenarios, except for posterior subgingival preparation design. There was a significant association between the dentist’s age and the selection of material and luting agent (*p* < 0.05) in all clinical scenarios, except for the luting agent selection in the posterior subgingival preparation designs (*p* < 0.05). Other nonclinical factors yielded mixed results; some preparation designs showed significant differences, while others did not, depending on the clinical scenario. **Conclusions:** Reliance on new materials and luting agents that require minimally invasive treatment with dental ceramics and resin cement is increasing. However, the choice of materials and luting agents is influenced by clinical presentation and nonclinical factors, making it crucial for dentists to be aware of these factors when selecting materials for single-crown restorations. **Clinical Implications:** An overall trend was observed for the use of strong monolithic ceramics with adhesive resin cements. These findings could assist dentists in reviewing and re-evaluating material choices in their clinical practices, both at a national and regional level. Additionally, the findings could be useful for dental policy makers, wholesale suppliers, and retail distributors in making future decisions.

## 1. Introduction

Advances in dental ceramics and luting agents have expanded dentists’ options for single-crown restorations [1]. The material selection for single complete-coverage crowns is influenced by a range of **clinical** and **nonclinical** factors [2,3,4,5]. **Clinical factors** are patient-specific or tooth-specific conditions that directly impact treatment decisions, such as tooth position (anterior or posterior), margin location (supragingival or subgingival), and preparation design for resistance and retention form. **Nonclinical factors** pertain to the professional and environmental context in which the clinician operates, such as practice location [6] and clinician specialization and experience [5].

Traditionally, full-metal and porcelain-fused-to-metal (PFM) crowns were the primary choices for single-crown restorations due to their strength and the acceptable esthetics of PFM crowns [6,7]. However, zirconia-based restorations, including monolithic and layered zirconia, have become the materials of choice for posterior restorations due to their mechanical properties, despite having some disadvantages, such as the opacity of monolithic zirconia and veneer chipping in layered zirconia [8,9,10]. Lithium disilicate ceramics are also widely employed for single-crown fabrication, given their versatility, allowing cementing or bonding to the tooth structure depending on the material’s thickness and other factors [11]. Although feldspathic and leucite-reinforced glass ceramics offer acceptable esthetics, they are mainly used for anterior teeth [12]. A Cochrane review [13] reported no significant advantage of one material over another in the case of single crowns, suggesting that material selection can be guided by the dentists’ experience and clinical circumstances. With diverse options for different clinical situations [14], practice settings [15], and individual experiences, dentists’ material preferences vary. For posterior teeth, zirconia-based crowns were favored as the restorative material in one study (32%) [5], whereas another study identified lithium disilicate, including zirconia-reinforced lithium silicate (ZLS), as the preferred material for posterior single crowns with supragingival margin preparation designs (45.2–52.2%) [4].

The choice of luting agents is another crucial factor when restoring a single crown. Traditionally, zinc phosphate, zinc polycarboxylate [16,17], and glass ionomer [18] cements were used for full-metal and PFM crowns, relying on preparation design for retention. However, these agents do not enhance esthetics [19], as they are typically used under opaque crown or core materials. Conversely, resin cement relies on adhesion and the formation of a hybrid layer on the tooth’s structure to enhance retention [20]. The selection of resin cements can impact the esthetics of single crowns, particularly when used with translucent ceramics such as feldspathic, leucite-reinforced, or lithium disilicate ceramics [21]. The choice between adhesive resin and other cements for luting single crowns continues to lack consensus among dentists. Although cementing with nonadhesive resin cements is quicker and less technically demanding, its retention depends on the preparation design, whereas luting with adhesive resin cements is technique-sensitive, especially for posterior teeth, due to isolation challenges and difficult accessibility [22]. Dentists select luting agents based on various factors and have been reported to adhesively bond single crowns in excessive occlusal reduction cases, on anterior teeth, or when using glass ceramics [23].

Dentistry in the State of Kuwait is rapidly evolving. Each year, new dentists join the workforce, many of whom are trained in countries such as the USA, UK, Egypt, India, and Saudi Arabia [24,25]. The introduction of dental insurance schemes catering to diverse segments of the population [26], coupled with an increase in the number of private dental clinics, has substantially reshaped dental care provision in Kuwait, moving away from government-run public health clinics toward a hybrid dental care system. It is reasonable to assume that this diversity in workforce and clinic settings influences dentists’ choices regarding materials and luting agents for single-crown restorations.

Previous studies have explored crown material and luting agent selection for single crowns independently, although the luting agent choices are interdependent on the crown material used, and their joint assessment is merited [4,5]. Prior survey-based studies primarily focused on establishing dentists’ preferences without providing suitable visual aids alongside the questions, potentially resulting in perception discrepancies among participating dentists. To the best of our knowledge, surveys of dentists’ preferences regarding crown materials and luting agents for single crowns are lacking in the Middle East region, particularly in the State of Kuwait. Furthermore, we are unaware of any relevant investigation in which survey questionnaires were supplemented with photographs depicting various clinical scenarios to enhance understanding and clarity.

This study aimed to investigate the choices made by dental practitioners in Kuwait when selecting materials and luting agents for anterior and posterior single complete coverage crowns across three different preparation designs: the ideal (equigingival cervical finish line and adequate abutment height), subgingival (adequate abutment height and subgingival cervical finish line), and short (reduced abutment height and equigingival cervical finish line) designs. Additionally, this study aimed to assess the influence of dentists’ age, specialty, clinical experience, practice type, and single crowns delivered per month using specific crown materials and luting cements for the three anterior and three posterior preparation designs. The null hypothesis asserts that there was no difference in the choice of restorative materials and luting cements among clinician groups, based on age, specialty, clinical experience, practice type, or the average number of single crowns cemented per month, across the three preparation design variations for both anterior and posterior teeth.

## 2. Materials and Methods

**Figure 1:**
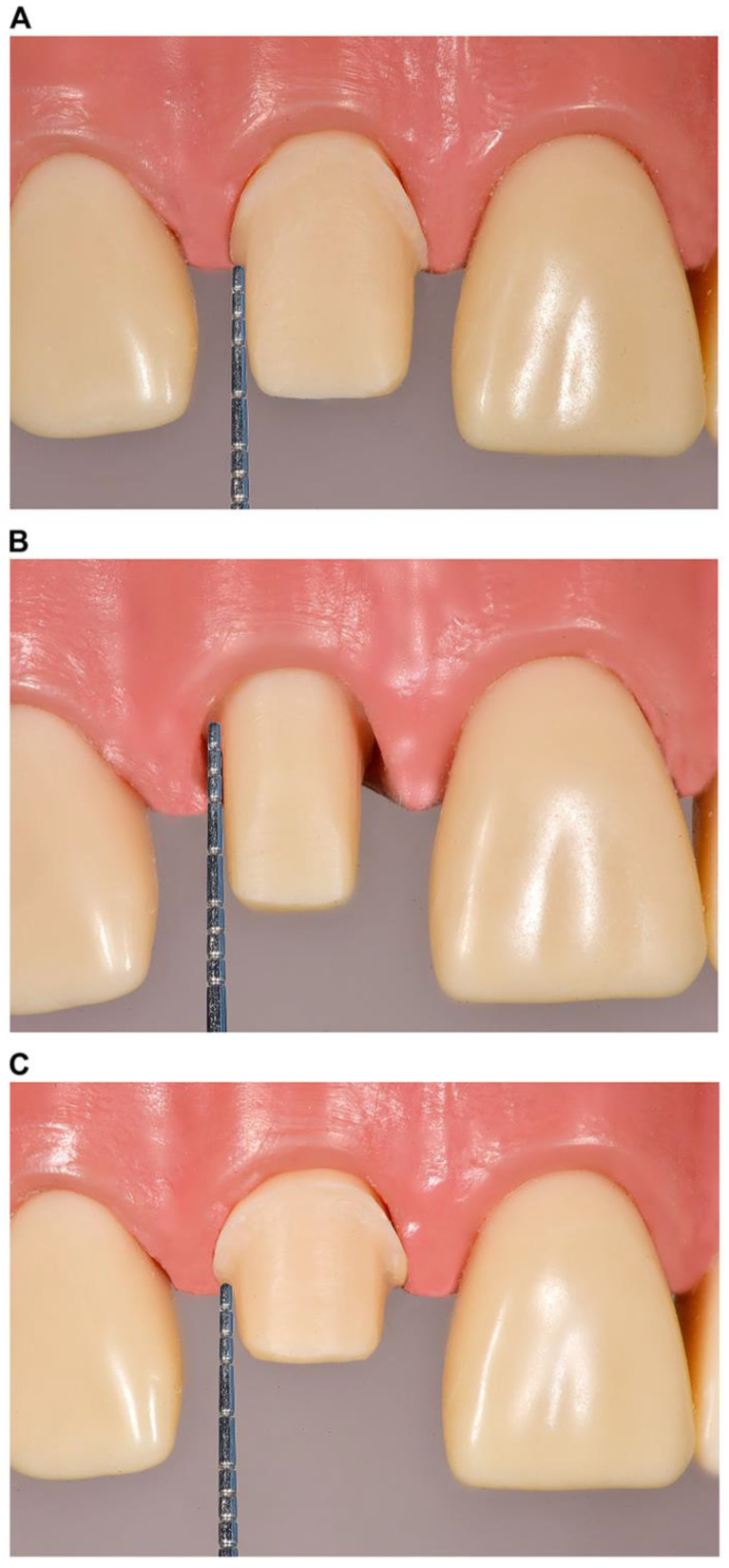
Illustration of single-crown preparation designs for the (**A**) supragingival or equigingival margin with an adequate preparation design height (≥3.0 mm); (**B**) subgingival margin with an adequate preparation design height (≥3.0 mm); and (**C**) short preparation designs (<3.0 mm) with supragingival or equigingival margins on the maxillary central incisor.

**Figure 2:**
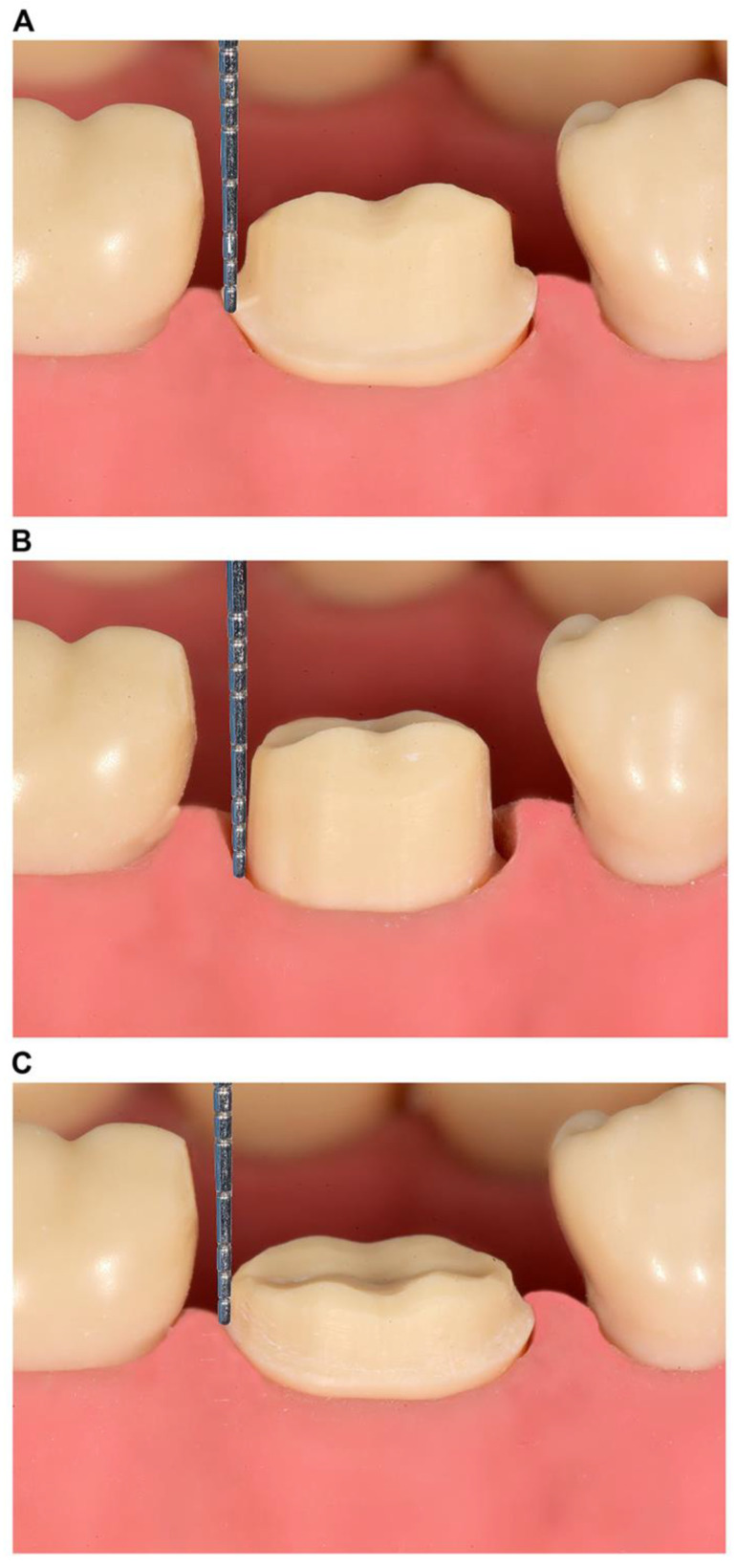
Illustration of single-crown preparation design for the (**A**) supragingival or equigingival margin with an adequate preparation design height (≥4.0 mm); (**B**) subgingival margin with an adequate preparation design height (≥4.0 mm); and (**C**) short preparation designs (<4.0 mm) with supragingival or equigingival margins on the mandibular first molar.

Approval for this study was obtained from the Ethical Committee of the Health Sciences Center, Kuwait University (VDR/EC-345), and the Ethical Committee of the Ministry of Health, Kuwait (approval no. 2023/2219; approved on 23 January 2023). Three single-crown preparation designs were simulated using Ivorine teeth (Frasaco GmbH, Tettnang, Germany), representing a right maxillary central incisor and a right mandibular first molar in a dentate typodont model. The clinical scenarios were as follows: (1) supragingival or equigingival margin with an adequate preparation design height (≥3 and ≥4 mm for the anterior and posterior teeth, respectively) (Figure 1A and Figure 2A); (2) subgingival margin, with a preparation design margin 1–2 mm below the free gingival margin and an adequate preparation design height (identical to scenario 1) (Figure 1B and Figure 2B); and (3) short preparation design (<3 and <4 mm for the anterior and posterior teeth, respectively) with a supragingival or equigingival margin (Figure 1C and Figure 2C). All preparation designs were photographed using a DSLR Nikon D7200 (Nikon Corporation, Tokyo, Japan), and the images were enhanced using digital software (Photoshop CC, Adobe, San Jose, CA, USA) by overlaying an accurately measured picture of a periodontal probe (XP 17/W, Hu-Friedy, Chicago, IL, USA) (Figure 1 and Figure 2).

Three dental clinicians with different backgrounds (a prosthodontist, a public health dentist, and a general dentist) developed and adapted a clinical scenario-based questionnaire based on similar questionnaires used in previous studies [2,4,5,23]. This questionnaire was used to explore the dentists’ choices of materials and luting agents for single-crown restorations. The questionnaire comprised two parts: demographic information collection, including age, gender, specialty, years of experience, practice type, practice governance location, and average number of single crowns delivered per month (Supplementary S1); and the presentation of six clinical scenario images (Figure 1 and Figure 2) with two multiple-choice questions each related to an image and regarding preferred materials and luting agents. The participants were advised to choose only one answer for 12 questions in total (Supplementary S1). The questionnaire underwent pilot testing with eight dentists before its distribution to the entire participant group. Based on the pilot study feedback, the questionnaire was slightly adjusted to refine the English language.

Between February 2023 and August 2023, the questionnaire was distributed by two general dentists (SA and AA) to dentists who regularly restored teeth using single-crown restorations at Kuwait Ministry of Health (MoH) specialty centers, private practices, and teaching faculty practice clinics (Kuwait University and Kuwaiti Board of Advanced General Dentistry). Informed consent was obtained from all participants. Completed survey forms were securely stored together until data collection was completed.

A power analysis was performed (G*power version 3.1, Franz Faul Universitat, Kiel, Germany) to determine the sample size. Based on the variable “years of clinical experience”, the sample size required to detect a 20% difference in the preference for using ceramic crowns for supragingival and subgingival preparation design finish lines [4] (80% power and 5% margin error) was estimated to be 241; this was then rounded up to 250 dentists. Descriptive statistics, including counts and percentages, were used to present dentist and practice characteristics. Cross-tabulations between restorative material and cement types, as well as comparisons between crown material/cement choices and dentist/practice characteristics, were performed. Significant differences were determined using the chi-square test. Data analysis was performed using SPSS (version 26, IBM, Armonk, NY, USA) with a 0.05 level of significance.

## 3. Results

**Figure 3:**
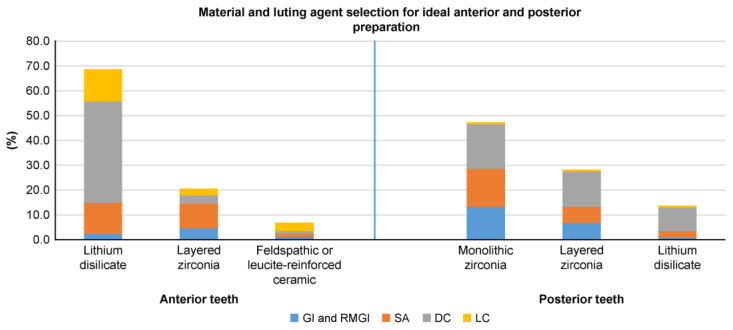
Dentist preferences (%) for materials and luting agents in supragingival or equigingival margins with an adequate preparation design height (≥3.0 mm) for anterior and posterior teeth.

**Figure 4:**
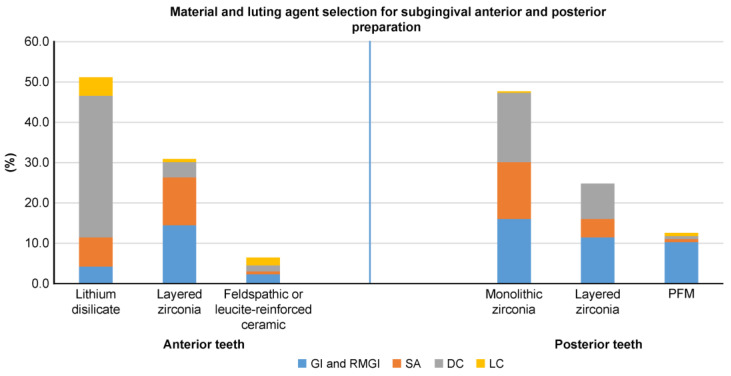
Dentist preferences (%) for materials and luting agents in subgingival margins with an adequate preparation design height (≥3.0 mm) for anterior and posterior teeth.

**Figure 5:**
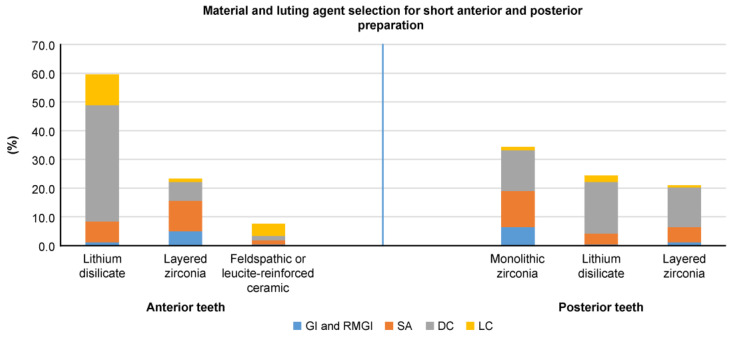
Dentist preferences (%) for materials and luting agents in short preparation designs (<3.0 mm) with supragingival or equigingival margins for anterior and posterior teeth.

In total, 300 survey forms were distributed, with 262 (87.3%) dentists completing the paper-based clinical scenario survey used to explore dentists’ preferences when selecting single-crown materials and luting agents for anterior and posterior teeth (Supplementary S2). Table 1 shows the participants’ demographics. Figure 3, Figure 4 and Figure 5 depict the percentages of materials and luting agents selected by the dentists for the three scenarios concerning both anterior and posterior teeth.

Supplementary Tables S1–S6 show the frequencies and percentages of materials and luting agents chosen by different dentist groups across the three scenarios based on the selected variables.

Lithium disilicate emerged as the top choice for all anterior tooth preparation designs, followed by layered zirconia and glass ceramic. For posterior teeth, monolithic zirconia was the favored material in all three scenarios, followed by layered zirconia in ideal and subgingival preparation designs and lithium disilicate for short preparation designs (Figure 3, Figure 4 and Figure 5). Dual-cure resin cement was predominantly favored across all preparation designs for anterior and posterior teeth, except for posterior subgingival preparation designs, in which GI and RMGI were equally preferred as the primary luting agents.

Among the examined factors, age significantly affected material and luting agent selection (*p* < 0.05) irrespective of the tooth location (anterior or posterior) or preparation design (ideal, subgingival, or short) (Supplementary Tables S1–S6). The only exception was for cement selection in posterior subgingival preparation designs, in which age had no significant effect (*p* < 0.05) (Supplementary Table S4). The variable “Specialty” (prosthodontist, general dentist, and others) significantly affected material selection for short anterior and posterior teeth (Supplementary Table S5) and subgingival posterior teeth preparation designs (*p* < 0.05) (Supplementary Table S3), but not for ideal preparation designs (Supplementary Table S1). Specialty did not significantly influence luting agent selection for any clinical scenario (*p* < 0.05), except for anterior ideal teeth preparation designs (Supplementary Table S2). The practice type (MoH, private practice, educational institution, and multiple affiliations) significantly impacted the selection of both material and luting agents for ideal anterior and posterior preparation designs (Supplementary Tables S1 and S2) and material selection for posterior subgingival (Supplementary Table S3) and anterior short preparation designs (Supplementary Table S5). However, the variable “years of experience” did not significantly affect material or cement choices across the entire surveyed cohort (*p* < 0.05; Supplementary Tables S1–S6). Other factors (number of delivered crowns and practice location) yielded mixed results, with some preparation designs exhibiting significant differences while others did not (Supplementary Tables S1–S6).

## 4. Discussion

The aim of this study was to evaluate dentists’ preferences for single-crown materials and luting cement and to investigate the factors influencing these selections for complete coverage crowns across various clinical scenarios. The null hypothesis stated that there would be no differences in the restorative material and luting cement choices among different clinician groups based on age, specialty, clinical experience, practice type, and the average number of single crowns cemented per month across the three preparation designs (for both anterior and posterior teeth). The dentists’ age significantly affected their choice of crown material and luting cement in all three clinical scenarios concerning both anterior and posterior teeth, except for the selection of cement for subgingival posterior teeth. Hence, this aspect of the null hypothesis was rejected. Specialty, practice type, and the number of single crowns delivered per month partially influenced material choices in certain scenarios for both anterior and posterior teeth, resulting in the partial rejection of the null hypothesis. The only factor showing no influence on material choices among the groups was the dentists’ years of experience, supporting the null hypothesis for this factor.

This study’s findings indicate that dental ceramics were the predominant material chosen for crown fabrication for both anterior and posterior single-unit crowns. Regarding anterior teeth, in all three scenarios, lithium disilicate emerged as the preferred material choice, followed by layered zirconia and glass ceramics. Regarding posterior teeth, monolithic zirconia was the top material choice in all scenarios, with variations in the second and third choices for each scenario depending on the clinical situation. For ideal posterior preparation designs, layered zirconia was the second most selected option, followed by lithium disilicate, whereas in short preparation designs, lithium disilicate was favored second, followed by layered zirconia. The preference for lithium disilicate—an etchable and bondable ceramic [27]—in short preparation designs may be attributed to the dentist’s reliance on adhesion for retention [28], although evidence suggests that zirconia-based materials can also be bonded [29]. In posterior subgingival preparation designs, dentists opted for PFM more frequently compared to ideal and short preparation designs; this is potentially attributed to challenges related to isolation and the use of simplified cementation protocols for PFM crown delivery to facilitate easier postcementation cleaning.

The findings of the present study reflect trends observed in other countries in which ceramic materials are preferred over metal-based restorations [4,5]; this is likely due to dentists’ preferences for ceramic materials as a more esthetic and cost-effective alternative to restorations based on noble metal alloys. A national practice-based survey in the US found that, for anterior tooth preparation designs, dentists favored lithium disilicate (54%), followed by layered zirconia (17%) and leucite-reinforced glass ceramics (13%), irrespective of the margin location. In posterior preparation designs, zirconia (32%) was the top choice, followed closely by PFM (31%) and then lithium disilicate (21%) [5]. In a German questionnaire-based study considering tooth and margin locations, dentists mainly selected lithium disilicate, including ZLS, for anterior supragingival preparation (59.7%) and subgingival preparation designs (48.5%), followed by layered zirconia (28.9% and 43.0%, respectively). For two posterior supragingival preparation designs involving mandibular and maxillary first molars, German dentists favored lithium disilicate, including ZLS, as the primary material (45.2% and 52.2%, respectively), followed by monolithic zirconia. Notably, for posterior subgingival preparation designs, a higher proportion of German dentists selected PFM as the material of choice [4].

We considered tooth and margin locations across three clinical scenarios; nevertheless, we observed a consistent preference among dentists for anterior teeth material selection relative to prior studies, with lithium disilicate emerging as the most selected material, followed by layered zirconia. This likely reflects the versatility of these materials and their applicability across various clinical scenarios involving anterior teeth. For posterior teeth, irrespective of the margin location, monolithic zirconia was most preferred, aligning with preferences among US dentists but differing from German dentists, who favored lithium disilicate, including ZLS for supragingival margins. Conversely, a higher proportion of German dentists selected PFM for subgingival margins. This variability in posterior material selection based on the clinical scenario suggests a departure from the ideal preparation design, with PFM and lithium disilicate being more common in posterior subgingival preparation and posterior short preparation designs, respectively, as discussed earlier.

Our study revealed dentists’ preferences for adhesive resin cement over water-based conventional cements, with dual-cure resin cement emerging as the preferred luting agent for both anterior teeth and posterior single crowns across all preparation designs. However, for subgingival posterior tooth preparation designs, a combination of glass ionomer and Resin-modified glass ionomer (RMGI) cements was favored over dual-cure resin cement (38.9% vs. 35.1%; Figure 4). The preference for dual-cure resin cement could be attributed to the corresponding preference for ceramic materials, requiring adhesion for retention. Although zirconia-based and lithium disilicate crowns can be luted with RMGI and self-adhesive resin cements, dentists in our study preferred dual-cure adhesive resin cements. This preference could be attributed to the versatility of dual-cure resin cement, with its self-curing mode rendering it ideal for opaque crown materials, such as zirconia-based restorations, and its color stability and light-cure mode rendering it an appropriate choice for anterior ceramic restorations. This versatility also simplifies inventory management in dental practices, making it more practical and efficient for dental teams to use this cement in various clinical scenarios. Finally, material selection and purchasing decisions are typically carried out centrally; individual dentists’ choices may be limited, forcing them to use only what the system provides.

These findings contrast with a national dental practice-based research study reporting that 39.1% of surveyed dentists had never bonded a crown, with US dentists preferring glass ionomer and RMGI cement 60.4% of the time when providing a single crown [23]. In Germany, a survey study focusing solely on the material type reported that dentists preferred adhesive cementation based on restorative materials, with only 2.8% using alloy-PFM-based materials and 77.9% using feldspathic/leucite-reinforced glass ceramics, excluding self-adhesive cement [2]. We attribute differences in luting agent preference between studies to the timing of data collection, with the US studies conducted in 2016–2017, whereas the German study was conducted in 2019–2020. We speculate that this shift in luting agent preference over time reflects a broader trend in the profession toward adhesive-type luting agents.

Alongside clinical considerations, we identified nonclinical factors associated with material and luting agent selection, including dentist-related and practice-related factors. Age, a dentist-related factor, significantly influenced single-crown material and luting agent selection across all clinical scenarios, except for luting agent selection in posterior subgingival preparation designs. A similar trend was reported in another study, in which the “years of experience” factor was significantly associated with material selection [23]. We attribute this to younger dentists being trained with advanced ceramics and luting agents or seasoned dentists developing preferences for newer materials and agents over time. The practice type, a practice-related factor, significantly impacted material and luting agent selection in ideal anterior and posterior preparation designs. This may be linked to the availability of materials and luting agents in certain clinical settings; some MoH specialty centers have transitioned from fabricating metal-based restorations to using zirconia-based alternatives for posterior teeth to expedite dental services. Another study reported that practice business may influence material selection, with busy practices opting for less technique-sensitive materials and less time-consuming luting techniques [5].

The number of single crowns delivered per month, a dentist-related and practice-related factor, significantly influenced material and luting agent selection in anterior tooth preparation designs, except for luting agent selection in subgingival preparation designs. Data indicate that dentists delivering > 20 single-unit crowns per month tend to select lithium disilicate as the material of choice for anterior teeth and prefer dual-cure resin cement as the luting agent. We speculate that dentists in such practices primarily focus on delivering esthetic dentistry, often involving multiple anterior teeth per patient. Specialty was significantly associated with material selection in short anterior and posterior preparation designs, as well as subgingival posterior preparation designs. Regarding luting agent selection, specialty was only statistically significant in the anterior ideal preparation design. Another study reported that specialty significantly influenced luting agent selection, with prosthodontists showing a preference for conventional cementation for polycrystalline ceramics and adhesive cementation for computer-aided design and manufacturing (CAD/CAM) resin composites compared to dentists specialized in conservative dentistry [2]. These findings suggest that nonclinical factors, including dentist-related and practice-related factors, influence material and luting agent selection in certain clinical scenarios.

Although this investigation covered three distinct clinical scenarios, certain clinical factors that could have potentially influenced dentists’ choices, such as interocclusal clearance for restoration thickness and pulp vitality status, were not considered, constituting a limitation of this study. Another limitation was the exclusion of newer generations of CAD/CAM materials due to their limited availability in the local market, thereby restricting dentists’ options. Dual-cure resin cement polymerization can be initiated by either light-cure activation or self-cure mode. However, this study did not include inquiries regarding the mode of activation or the adhesive protocol for dual-cure cement, both of which are factors that impact restoration success [30,31]. This study relied on self-reported data, which may reflect idealized rather than actual clinical practices, representing a potential limitation.

Based on our findings, a general trend was observed concerning the reliance on strong monolithic ceramics with adhesive resin cements. We anticipate a growing reliance on dental ceramics as the material of choice for single-crown restoration, with a further reduction in the use of metal-based restorations in Kuwait. Additionally, our results suggest a shift toward adhesive resin cement as a luting agent for single crowns, accompanied by the decreased use of acid–base cements. These findings could assist dentists in re-evaluating their current material choices with respect to different clinical scenarios, both at a national and regional level. Dental policy makers and dental suppliers can use these findings to make future decisions regarding material choices.

These findings indicate a trend toward minimally invasive treatment, as more dentists prefer using adhesive luting agents that require conservative tooth preparation designs with less emphasis on retention and resistance form. With the advancement of newer CAD/CAM restorative materials, direct training and continuing education courses specifically addressing optimal adhesive protocols for current materials are essential. Understanding current material selection and associated factors will facilitate the accommodation and implementation of future advancements in the field. It is imperative to investigate and document future trends, including the influence of hybrid ceramics, nanoceramics, and 3D-printed materials on material selection for single crowns. Furthermore, similar surveys should be conducted to ascertain the preferences of dentists and specialists for other types of dental prostheses, including onlays, inlays, veneers, and partial fixed dental prostheses, in order to better understand current national, regional, and international trends.

## 5. Conclusions

This study reveals that dentists’ selections for single-crown materials and luting agents are influenced by clinical factors such as tooth location and margin position. Moreover, for nonclinical factors, the dentist’s age was found to be a significant factor in the choice of materials. Monolithic zirconia was the predominant choice for posterior teeth, and lithium disilicate was the predominant choice for anterior teeth, with both exhibiting clinical versatility and esthetic value. Dual-cure resin cement was the predominant luting agent, probably due to its practical benefits and compatibility with ceramic restorations. However, perhaps due to the challenges associated with isolation during the cementation of subgingival posterior restorations, conventional cements—such as glass and resin-modified glass ionomers—were preferred. Recognizing these influential factors is important for adapting clinical practices effectively and staying current with evolving trends in minimally invasive ceramic restorations and adhesive cementation techniques. These findings may aid dentists in reassessing their material selections in clinical practices at both national and regional levels. They may also assist dental policymakers, wholesale suppliers, and retail distributors in making future decisions regarding material selection. Moreover, future studies should evaluate other influencing clinical factors, newly developed restorative materials, and larger geographic contexts. Our results highlight the necessity of continuous education and training for dentists so that their material selection and the luting procedures used align with the most recent research findings and changing clinical standards.

## Figures and Tables

**Table 1:** Demographic characteristics of study participants.

Variable		N (%)
Age	20–30	26 (9.9)
	31–40	151 (57.6)
	41–50	67 (25.6)
Gender	>51	18 (6.9)
	Male	163 (62.2)
	Female	99 (37.8)
Specialty	General dentist	89 (34.0)
	Prosthodontist	141 (53.8)
	Others	32 (12.2)
Experience	≤10	113 (43.1)
	11–15	80 (30.5)
	≥16	69 (26.3)
Practice type	Ministry of Health	105 (40.1)
	Private clinic	54 (20.6)
	Teaching institute	20 (7.6)
	Multiple places	83 (31.7)
Governorate	Jahra	45 (17.2)
	Farwaniya	34 (13.0)
	Asimah	54 (20.6)
	Ahmadi	37 (14.1)
	Hawally	82 (31.3)
	>1 governorate	10 (3.8)
Number of single crowns delivered per month	<10	53 (20.2)
	10–20	93 (35.5)
	>20	116 (44.3)

## Data Availability

The datasets used and/or analyzed during the current study are available from the corresponding author upon reasonable request.

## Supplementary Materials

The following supporting information can be downloaded at https://www.mdpi.com/article/10.3390/dj13050207/s1. Table S1: Number and percentage of answers for single-unit crown materials in supragingival or equigingival margins with adequate preparation design heights (≥3.0 mm) for anterior and posterior teeth according to age, specialty, years of experience, number of single crowns delivered per month, and practice type. Table S2: Number and percentage of answers for single-unit crown luting agents in supragingival or equigingival margins with adequate preparation design heights (≥3.0 mm) for anterior and posterior teeth according to age, specialty, years of experience, number of single crowns delivered per month, and practice type. Table S3: Number and percentage of answers for single-unit crown materials in the subgingival margin with adequate preparation design heights (≥3.0 mm) for anterior and posterior teeth according to age, specialty, years of experience, number of single crowns delivered per month, and practice type. Table S4: Number and percentage of answers for single-unit crown luting agents in the subgingival margin with adequate preparation design height (≥3.0 mm) for anterior and posterior teeth according to age, specialty, years of experience, number of single crowns delivered per month, and practice type. Table S5: Number and percentage of answers for single-unit crown materials in short preparation designs (<3.0 mm) with supragingival or equigingival margins for anterior and posterior teeth according to age, specialty, years of experience, number of single crowns delivered per month, and practice type. Table S6: Number and percentage of answers for single-unit crown luting agents in short preparation designs (<3.0 mm) with supragingival or equigingival margins for anterior and posterior teeth according to age, specialty, years of experience, number of single crowns delivered per month, and practice type. Supplementary S1: Questionnaire. Supplementary S2: Material and luting agent selection for anterior and posterior teeth.

## Abbreviations

The following abbreviations are used in this manuscript:

MoH	Ministry of Health
PFM	Porcelain-fused-to-metal
ZLS	Zirconia-reinforced lithium silicate
CAD/CAM	Computer-aided design (CAD)/computer-aided manufacturing (CAM)
