# Supplementary material for: Factors Influencing the Selection of Materials and Luting Agents for Single-Crown Restorations
Source: Dent J (Basel). 2025 May 9;13(5):207. doi: 10.3390/dj13050207 (PMC12109942; doi:10.3390/dj13050207)
Supplement: Supplementary file 1 [file dentistry-13-00207-s001.zip › Supplementary S1.pdf]

Selection of Materials and Luting Agents for Single Crown Restorations:
A National Survey of Dentists

Demographics

Age (years)

- 20–30
- 31–40
- 41–50
- 51–60
- > 60

Gender

- Male
- Female

Specialty

- General dentist
- Prosthodontist
- Other (please specify) _____________________

Years of experience

- 1–5
- 6–10
- 11–15
- 16–20
- > 20

Practice type; check all that apply

- Ministry of Health
- Private
- Educational institution
- Other (please specify)________________________

Governate

- Jahra
- Farwaniya
- Asimah
- Ahmadi
- Hawally

Number of single crowns delivered per month, on average

- Fewer than 10
- 10–20
- More than 20

	SUPRA OR EQUI-GINGIVAL MARGIN WITH ADEQUATE PREPARATION HEIGHT (≥ 3.0 mm)	SUBGINGIVAL MARGIN WITH ADEQUATE PREPARATION HEIGHT (≥ 3.0 mm)	SHORT PREPARATION (3.0 mm >) WITH SUPRA OR EQUI-GINGIVAL MARGIN
**CLINICAL SCENARIO**	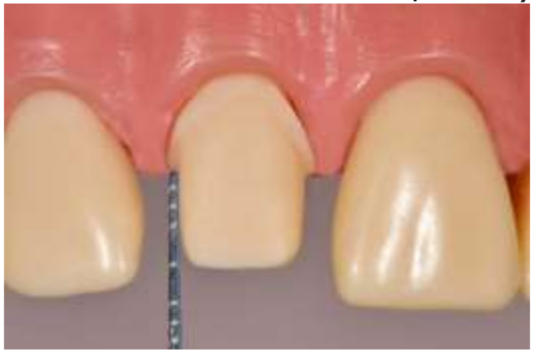	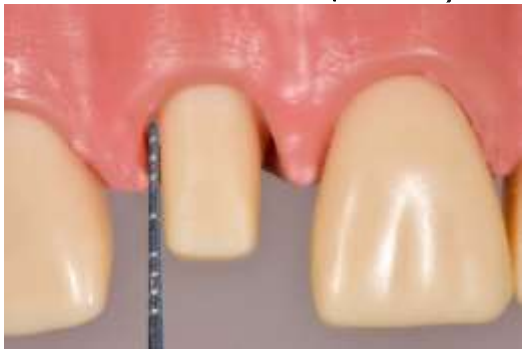	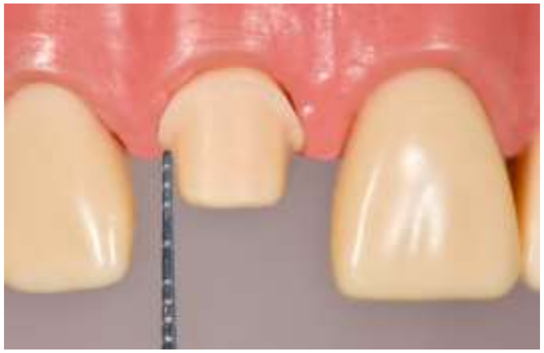
**WHAT IS YOUR PREFERRED RESTORATION MATERIAL FOR THIS CLINICAL SCENARIO?** (ONLY ONE ANSWER **)**	o Full-metal<br>o Ceramo-metal, Porcelain-Fused-to-metal (PFM)<br>o Feldspathic or leucite-reinforced ceramic<br>o Lithium disilicate<br>o Monolithic zirconia<br>o Layered zirconia<br>o Other (please specify or state the brand name)	o Full-metal<br>o Ceramo-metal, Porcelain-Fused-to-metal (PFM)<br>o Feldspathic or leucite-reinforced ceramic<br>o Lithium disilicate<br>o Monolithic zirconia<br>o Layered zirconia<br>o Other (please specify or state the brand name)	o Full-metal<br>o Ceramo-metal, Porcelain-Fused-to-metal (PFM)<br>o Feldspathic or leucite-reinforced ceramic<br>o Lithium disilicate<br>o Monolithic zirconia<br>o Layered zirconia<br>o Other (please specify or state the brand name)
**WHAT TYPE OF LUTING CEMENT WOULD YOU USE IN THIS CLINICAL CONDITION?** (ONLY ONE ANSWER **)**	o Zinc Phosphate cement<br>o Carboxylate cement (Durealon)<br>o Glass ionomer (GC Fuji Plus, Ketac Cem)<br>o Resin-Modified Glass Ionomer RMGI (RelyX Luting 2, GC FujiCem 2)<br>o Self-adhesive cement (RelyX Unicem, G-CEM One, SpeedCem)<br>o Dual-cure resin cement (G-CEM LinkForce, RelyX Ultimate, Variolink Esthetics DC)<br>o Light cure resin cement (RelyX Veneer, Variolink Esthetics LC)<br>o Other(please specific or state the brand)	o Zinc Phosphate cement<br>o Carboxylate cement (Durealon)<br>o Glass ionomer (GC Fuji Plus, Ketac Cem)<br>o Resin-Modified Glass Ionomer RMGI (RelyX Luting 2, GC FujiCem 2)<br>o Self-adhesive cement (RelyX Unicem, G-CEM One, SpeedCem)<br>o Dual-cure resin cement (G-CEM LinkForce, RelyX Ultimate, Variolink Esthetics DC)<br>o Light cure resin cement (RelyX Veneer, Variolink Esthetics LC)<br>o Other(please specific or state the brand)	o Zinc Phosphate cement<br>o Carboxylate cement (Durealon)<br>o Glass ionomer (GC Fuji Plus, Ketac Cem)<br>o Resin-Modified Glass Ionomer RMGI (RelyX Luting 2, GC FujiCem 2)<br>o Self-adhesive cement (RelyX Unicem, G-CEM One, SpeedCem)<br>o Dual-cure resin cement (G-CEM LinkForce, RelyX Ultimate, Variolink Esthetics DC)<br>o Light cure resin cement (RelyX Veneer, Variolink Esthetics LC)<br>o Other(please specific or state the brand)

	SUPRA OR EQUI-GINGIVAL MARGIN WITH ADEQUATE PREPARATION HEIGHT (≥ 4.0 mm)	SUBGINGIVAL MARGIN WITH ADEQUATE PREPARATION HEIGHT (≥ 4.0 mm)	SHORT PREPARATION (4.0 mm >) WITH SUPRA OR EQUI-GINGIVAL MARGIN
**CLINICAL SCENARIO**	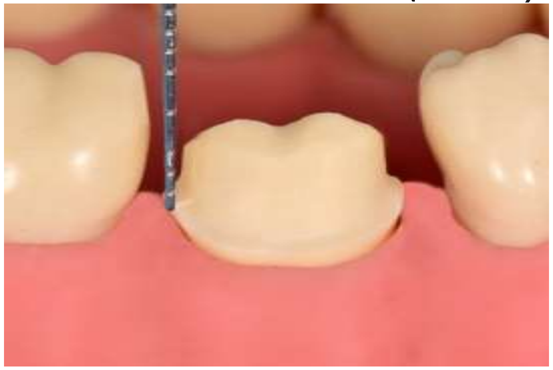	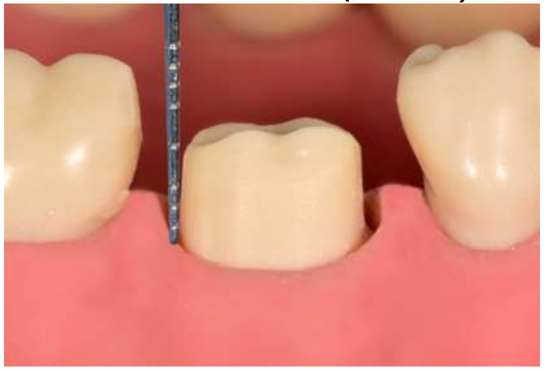	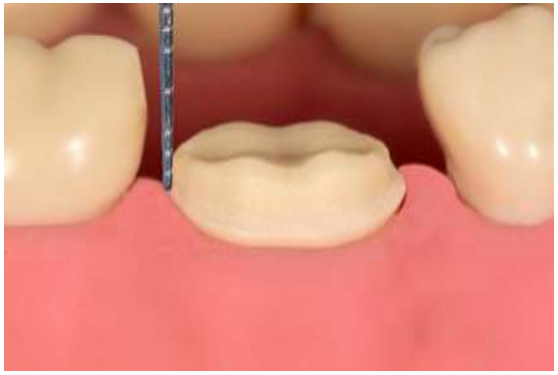
**WHAT IS YOUR PREFERRED RESTORATION MATERIAL FOR THIS CLINICAL SCENARIO?** (ONLY ONE ANSWER **)**	o Full-metal<br>o Ceramo-metal, Porcelain-Fused-to-metal (PFM)<br>o Feldspathic or leucite-reinforced ceramic<br>o Lithium disilicate<br>o Monolithic zirconia<br>o Layered zirconia<br>o Other (please specify or state the brand name)	o Full-metal<br>o Ceramo-metal, Porcelain-Fused-to-metal (PFM)<br>o Feldspathic or leucite-reinforced ceramic<br>o Lithium disilicate<br>o Monolithic zirconia<br>o Layered zirconia<br>o Other (please specify or state the brand name)	o Full-metal<br>o Ceramo-metal, Porcelain-Fused-to-metal (PFM)<br>o Feldspathic or leucite-reinforced ceramic<br>o Lithium disilicate<br>o Monolithic zirconia<br>o Layered zirconia<br>o Other (please specify or state the brand name)
**WHAT TYPE OF LUTING CEMENT WOULD YOU USE IN THIS CLINICAL CONDITION?** (ONLY ONE ANSWER **)**	o Zinc Phosphate cement<br>o Carboxylate cement (Durealon)<br>o Glass ionomer (GC Fuji Plus, Ketac Cem)<br>o Resin-Modified Glass Ionomer RMGI (RelyX Luting 2, GC FujiCem 2)<br>o Self-adhesive cement (RelyX Unicem, G-CEM One, SpeedCem)<br>o Dual-cure resin cement (G-CEM LinkForce, RelyX Ultimate, Variolink Esthetics DC)<br>o Light cure resin cement (RelyX Veneer, Variolink Esthetics LC)<br>o Other(please specific or state the brand)	o Zinc Phosphate cement<br>o Carboxylate cement (Durealon)<br>o Glass ionomer (GC Fuji Plus, Ketac Cem)<br>o Resin-Modified Glass Ionomer RMGI (RelyX Luting 2, GC FujiCem 2)<br>o Self-adhesive cement (RelyX Unicem, G-CEM One, SpeedCem)<br>o Dual-cure resin cement (G-CEM LinkForce, RelyX Ultimate, Variolink Esthetics DC)<br>o Light cure resin cement (RelyX Veneer, Variolink Esthetics LC)<br>o Other(please specific or state the brand)	o Zinc Phosphate cement<br>o Carboxylate cement (Durealon)<br>o Glass ionomer (GC Fuji Plus, Ketac Cem)<br>o Resin-Modified Glass Ionomer RMGI (RelyX Luting 2, GC FujiCem 2)<br>o Self-adhesive cement (RelyX Unicem, G-CEM One, SpeedCem)<br>o Dual-cure resin cement (G-CEM LinkForce, RelyX Ultimate, Variolink Esthetics DC)<br>o Light cure resin cement (RelyX Veneer, Variolink Esthetics LC)<br>o Other(please specific or state the brand)
