# Supplementary material for: Factors Influencing the Selection of Materials and Luting Agents for Single-Crown Restorations
Source: Dent J (Basel). 2025 May 9;13(5):207. doi: 10.3390/dj13050207 (PMC12109942; doi:10.3390/dj13050207)
Supplement: Supplementary file 1 [file dentistry-13-00207-s001.zip › Supplementary S2.pdf]

Supplementary S2
Anterior Teeth
Materials and luting agents selection

Table B1. Materials and luting agents selection for anterior teeth with supra or equi-gingival margin with adequate preparation height (≥ 3.0 mm).

	**Glass Ionomer & Resin-Modified Glass Ionomer**	**Self-adhesive cement**	**Dual-cure resin cement**	**Light cure resin cement**	**Other**	**Total**
**Lithium disilicate**	6 (3.3%)	33 (18.3%)	107 (59.4%)	34 (18.9%)	0 (0.0%)	180 (68.7%)
**Layered zirconia**	12 (22.2%)	26 (48.1%)	9 (16.7%)	7 (13.0%)	0 (0.0%)	54 (20.6%)
**Feldspathic or leucite-reinforced ceramic**	3 (16.7%)	3 (16.7%)	3 (16.7%)	9 (50.0%)	0 (0.0%)	18 (6.9%)
**Other**	6 (60.0%)	0 (0.0%)	2 (20.0%)	1 (10.0%)	1 (10.0%)	10 (3.8%)
**Total**	27 (10.3%)	62 (23.7%)	121 (46.2%)	51 (19.5%)	1 (0.4%)	262 (100.0%)

Table B2. Materials and luting agents selection for anterior teeth with subgingival margin with adequate preparation height (≥ 3.0 mm).

	**Glass Ionomer & Resin-Modified Glass Ionomer**	**Self-adhesive cement**	**Dual-cure resin cement**	**Light cure resin cement**	**Other**	**Total**
**Lithium disilicate**	11 (8.1%)	19 (14.0%)	92 (67.6%)	12 (8.8%)	2 (1.5%)	136 (51.9%)
**Layered zirconia**	38 (46.9%)	31 (38.3%)	10 (12.3%)	2 (2.5%)	0 (0.0%)	81 (30.9%)
**Feldspathic or leucite-reinforced ceramic**	6 (35.3%)	2 (11.8%)	4 (23.5%)	5 (29.4%)	0 (0.0%)	17 (6.5%)
**Other**	17 (60.7%)	4 (14.3%)	4 (14.3%)	1 (3.6%)	2 (7.1%)	28 (10.7%)
**Total**	72 (27.5%)	56 (21.4%)	110 (42.0%)	20 (7.6%)	4 (1.5%)	262(100.0%)

Table B3.: Materials and luting agents selection for anterior teeth with short preparation (< 3.0 mm) with supra or equi-gingival margin.

	**Glass Ionomer & Resin-Modified Glass Ionomer**	**Self-adhesive cement**	**Dual-cure resin cement**	**Light cure resin cement**	**Other**	**Total**
**Lithium disilicate**	3 (1.9%)	19 (12.2%)	106 (67.9%)	28 (17.9%)	0 (0.0%)	156 (59.5%)
**Layered zirconia**	13 (21.3%)	28 (45.9%)	17 (27.9%)	3 (4.9%)	0 (0.0%)	61 (23.3%)
**Feldspathic or leucite-reinforced ceramic**	1 (5.0%)	4 (20.0%)	4 (20.0%)	11 (55.0%)	0 (0.0%)	20 (7.6%)
**Other**	8 (32.0%)	7 (28.0%)	4 (16.0%)	2 (8.0%)	4 (16.0%)	25 (9.5%)
**Total**	25 (9.5%)	58 (22.1%)	131 (50.0%)	44 (16.8%)	4 (1.5%)	262 (100.0%)

Posterior Teeth
Materials and luting agents selection

Table B4. Materials and luting agents selection for posterior teeth supra or equi-gingival margin with adequate preparation height (≥ 3.0 mm).

	Glass Ionomer & Resin-Modified Glass Ionomer	Self-adhesive cement	Dual-cure resin cement	Light cure resin cement	Other	Total
**Monolithic zirconia**	35 (28.0%)	40 (32.0%)	47 (37.6%)	2 (1.6%)	1 (0.8%)	125 (47.7%)
**Layered zirconia**	18 (24.3%)	17 (23.0%)	37 (50.0%)	2 (2.7%)	0 (0.0%)	74 (28.2%)
**Lithium disilicate**	2 (5.6%)	7 (19.4%)	25 (69.4%)	2 (5.6%)	0 (0.0%)	36 (13.7%)
**Other**	12 (44.4%)	2 (7.4%)	10 (37.0%)	0 (0.0%)	3 (11.1%)	27 (10.3%)
**Total**	67 (25.6%)	66 (25.2%)	119 (45.4%)	6 (2.3%)	4 (1.5%)	262 (100.0%)

Table B5. Materials and luting agents selection for posterior teeth with subgingival margin with adequate preparation height (≥ 3.0 mm)

	Glass Ionomer & Resin-Modified Glass Ionomer	Self-adhesive cement	Dual-cure resin cement	Light cure resin cement	Other	Total
**Monolithic zirconia**	42 (33.1%)	37 (29.1%)	45 (35.4%)	1 (0.8%)	2 (1.6%)	127 (48.5%)
**Layered zirconia**	30 (46.2%)	12 (18.5%)	23 (35.4%)	0 (0.0%)	0 (0.0%)	65 (24.8%)
**Ceramo-metal, Porcelain-Fused-to-metal (PFM)**	27 (75.0%)	2 (5.6%)	2 (5.6%)	2 (5.6%)	3 (8.3%)	36 (13.7%)
**Other**	3 (8.8%)	7 (20.6%)	22 (64.7%)	0 (0.0%)	2 (5.9%)	34 (13.0%)
**Total**	102 (38.9%)	58 (22.1%)	92 (35.1%)	3 (1.1%)	7 (2.7%)	262 (100.0%)

Table B6.: Materials and luting agents selection for posterior teeth with short preparation (< 3.0 mm) with supra or equi-gingival margin.

	**Glass Ionomer & Resin-Modified Glass Ionomer**	**Self-adhesive cement**	**Dual-cure resin cement**	**Light cure resin cement**	**Other**	**Total**
**Monolithic zirconia**	17 (18.7%)	33 (36.3%)	37 (40.7%)	3 (3.3%)	1 (1.1%)	91 (34.7%)
**Lithium disilicate**	1 (1.6%)	10 (15.6%)	47 (73.4%)	6 (9.4%)	0 (0.0%)	64 (24.4%)
**Layered zirconia**	3 (5.5%)	14 (25.5%)	36 (65.5%)	2 (3.6%)	0 (0.0%)	55 (21.0%)
**Other**	25 (48.1%)	7 (13.5%)	13 (25.0%)	2 (3.8%)	5 (9.6%)	52 (19.8%)
**Total**	46 (17.6%)	64 (24.4%)	133 (50.8%)	13 (5.0%)	6 (2.3%)	262 (100.0%)
