# Supplementary material for: Factors Influencing the Selection of Materials and Luting Agents for Single-Crown Restorations
Source: Dent J (Basel). 2025 May 9;13(5):207. doi: 10.3390/dj13050207 (PMC12109942; doi:10.3390/dj13050207)
Supplement: Supplementary file 1 [file dentistry-13-00207-s001.zip › Supplementary Tables.pdf]

Table S1: Number and percentage of answers for single-unit crown materials in supragingival or equigingival margins with adequate preparation height (≥3.0 mm) for anterior and posterior teeth according to age, specialty, years of experience, number of single crowns delivered per month, and practice type.

		(PFM)		Feldspathic		Lithium disilicate		Monolithic zirconia		Layered zirconia		Other		Total		P value	
**Variable**		**Ant**	**Post**	**Ant**	**Post**	**Ant**	**Post**	**Ant**	**Post**	**Ant**	**Post**	**Ant**	**Post**	**Ant**	**Post**	**Ant**	**Post**
**Age**	**20-30**	2 (7.7%)	1 (3.8%)	3 (11.5%)	0 (0.0%)	15 (57.7%)	4 (15.4%)	1 (3.8%)	14 (53.8%)	5 (19.2%)	7 (26.9%)	0 (0.0%)	0 (0.0%)	26 (100.0%)	26 (100.0%)		
	**31-40**	0 (0.0%)	6 (4.0%)	8 (5.3%)	5 (3.3%)	114 (75.5%)	25 (16.6%)	3 (2.0%)	80 (53.0%)	25 (16.6%)	32 (21.2%)	1 (0.7%)	3 (2.0%)	151 (100.0%)	151 (100.0%)		
	**41-50**	0 (0.0%)	1 (1.5%)	6 (9.0%)	7 (10.4%)	41 (61.2%)	6 (9.0%)	0 (0.0%)	21 (31.3%)	20 (29.9%)	32 (47.8%)	0 (0.0%)	0 (0.0%)	67 (100.0%)	67 (100.0%)		
	**≥51**	0 (0.0%)	3 (16.7%)	1 (5.6%)	0 (0.0%)	10 (55.6%)	1 (5.6%)	2 (11.1%)	10 (55.6%)	4 (22.2%)	3 (16.7%)	1 (5.6%)	1 (5.6%)	18 (100.0%)	18 (100.0%)	<0.001	0.001
**Specialty**	**General Dentist**	2 (2.2%)	2 (2.2%)	7 (7.9%)	3 (3.4%)	60 (67.4%)	12 (13.5%)	3 (3.4%)	43 (48.3%)	17 (19.1%)	28 (31.5%)	0 (0.0%)	1 (1.1%)	89 (100.0%)	89 (100.0%)		
	**Prosthodontist**	0 (0.0%)	6 (4.3%)	10 (7.1%)	9 (6.4%)	95 (67.4%)	16 (11.3%)	3 (2.1%)	71 (50.4%)	31 (22.0%)	36 (25.5%)	2 (1.4%)	3 (2.1%)	141 (100.0%)	141 (100.0%)		
	**Others**	0 (0.0%)	3 (9.4%)	1 (3.1%)	0 (0.0%)	25 (78.1%)	8 (25.0%)	0 (0.0%)	11 (34.4%)	6 (18.8%)	10 (31.3%)	0 (0.0%)	0 (0.0%)	32 (100.0%)	32 (100.0%)	0.595	0.336
**Experience**	**≤10**	2 (1.8%)	6 (5.3%)	5 (4.4%)	1 (0.9%)	83 (73.5%)	18 (15.9%)	1 (0.9%)	60 (53.1%)	21 (18.6%)	26 (23.0%)	1 (0.9%)	2 (1.8%)	113 (100.0%)	113 (100.0%)		
	**10-15**	0 (0.0%)	1 (1.3%)	9 (11.3%)	5 (6.3%)	54 (67.5%)	13 (16.3%)	3 (3.8%)	39 (48.8%)	14 (17.5%)	21 (26.3%)	0 (0.0%)	1 (1.3%)	80 (100.0%)	80 (100.0%)		
	**≥16**	0 (0.0%)	4 (5.8%)	4 (5.8%)	6 (8.7%)	43 (62.3%)	5 (7.2%)	2 (2.9%)	26 (37.7%)	19 (27.5%)	27 (39.1%)	1 (1.4%)	1 (1.4%)	69 (100.0%)	69 (100.0%)	0.294	0.071
**Number of single crowns delivered per month**	**< 10**	2 (3.8%)	4 (7.5%)	2 (3.8%)	2 (3.8%)	38 (71.7%)	4 (7.5%)	2 (3.8%)	29 (54.7%)	8 (15.1%)	13 (24.5%)	1 (1.9%)	1 (1.9%)	53 (100.0%)	53 (100.0%)		
	**10-20**	0 (0.0%)	2 (2.2%)	5 (5.4%)	3 (3.2%)	53 (57.0%)	12 (12.9%)	2 (2.2%)	52 (55.9%)	32 (34.4%)	24 (25.8%)	1 (1.1%)	0 (0.0%)	93 (100.0%)	93 (100.0%)		
	**> 20**	0 (0.0%)	5 (4.3%)	11 (9.5%)	7 (6.0%)	89 (76.7%)	20 (17.2%)	2 (1.7%)	44 (37.9%)	14 (12.1%)	37 (31.9%)	0 (0.0%)	3 (2.6%)	116 (100.0%)	116 (100.0%)	0.001	0.231

**Practice Type**	**MOH**	2 (1.9%)	5 (4.8%)	3 (2.9%)	1 (1.0%)	77 (73.3%)	10 (9.5%)	2 (1.9%)	56 (53.3%)	21 (20.0%)	32 (30.5%)	0 (0.0%)	1 (1.0%)	105 (100.0%)	105 (100.0%)		
	**Private**	0 (0.0%)	1 (1.9%)	6 (11.1%)	4 (7.4%)	41 (75.9%)	14 (25.9%)	4 (7.4%)	16 (29.6%)	3 (5.6%)	17 (31.5%)	0 (0.0%)	2 (3.7%)	54 (100.0%)	54 (100.0%)		
	**Edu. Inst.**	0 (0.0%)	4 (20.0%)	3 (15.0%)	0 (0.0%)	12 (60.0%)	3 (15.0%)	0 (0.0%)	9 (45.0%)	5 (25.0%)	4 (20.0%)	0 (0.0%)	0 (0.0%)	20 (100.0%)	20 (100.0%)		
	**Multiple**	0 (0.0%)	1 (1.2%)	6 (7.2%)	7 (8.4%)	50 (60.2%)	9 (10.8%)	0 (0.0%)	44 (53.0%)	25 (30.1%)	21 (25.3%)	2 (2.4%)	1 (1.2%)	83 (100.0%)	83 (100.0%)	0.004	0.001

Table S2: Number and percentage of answers for single-unit crown luting agents in supragingival or equigingival margins with adequate preparation height (≥3.0 mm) for anterior and posterior teeth according to age, specialty, years of experience, number of single crowns delivered per month, and practice type.

		Glass ionomer		Resin-Modified Glass Ionomer		Self-adhesive cement		Dual-cure resin cement		Light cure resin cement		Other		Total		P value	
**Variable**		**Ant**	**Post**	**Ant**	**Post**	**Ant**	**Post**	**Ant**	**Post**	**Ant**	**Post**	**Ant**	**Post**	**Ant**	**Post**	**Ant**	**Post**
**Age**	**20-30**	3 (11.5%)	4 (15.4%)	2 (7.7%)	2 (7.7%)	2 (7.7%)	6 (23.1%)	12 (46.2%)	12 (46.2%)	7 (26.9%)	2 (7.7%)	0 (0.0%)	0 (0.0%)	26 (100.0%)	26 (100.0%)		
	**31-40**	3 (2.0%)	15 (9.9%)	7 (4.6%)	19 (12.6%)	35 (23.2%)	43 (28.5%)	77 (51.0%)	70 (46.4%)	29 (19.2%)	3 (2.0%)	0 (0.0%)	1 (0.7%)	151 (100.0%)	151 (100.0%)		
	**41-50**	2 (3.0%)	14 (20.9%)	6 (9.0%)	5 (7.5%)	23 (34.3%)	14 (20.9%)	23 (34.3%)	32 (47.8%)	13 (19.4%)	1 (1.5%)	0 (0.0%)	1 (1.5%)	67 (100.0%)	67 (100.0%)		
	**≥51**	2 (11.1%)	6 (33.3%)	2 (11.1%)	2 (11.1%)	2 (11.1%)	3 (16.7%)	9 (50.0%)	5 (27.8%)	2 (11.1%)	0 (0.0%)	1 (5.6%)	2 (11.1%)	18 (100.0%)	18 (100.0%)	0.002	0.013
**Specialty**	**General Dentist**	3 (3.4%)	8 (9.0%)	10 (11.2%)	6 (6.7%)	10 (11.2%)	19 (21.3%)	42 (47.2%)	52 (58.4%)	24 (27.0%)	2 (2.2%)	0 (0.0%)	2 (2.2%)	89 (100.0%)	89 (100.0%)		
	**Prosthodontist**	7 (5.0%)	24 (17.0%)	7 (5.0%)	19 (13.5%)	42 (29.8%)	40 (28.4%)	65 (46.1%)	54 (38.3%)	19 (13.5%)	2 (1.4%)	1 (0.7%)	2 (1.4%)	141 (100.0%)	141 (100.0%)		
	**Others**	0 (0.0%)	7 (21.9%)	0 (0.0%)	3 (9.4%)	10 (31.3%)	7 (21.9%)	14 (43.8%)	13 (40.6%)	8 (25.0%)	2 (6.3%)	0 (0.0%)	0 (0.0%)	32 (100.0%)	32 (100.0%)	0.011	0.14
**Experience**	**≤10**	5 (4.4%)	15 (13.3%)	5 (4.4%)	12 (10.6%)	33 (29.2%)	34 (30.1%)	53 (46.9%)	49 (43.4%)	17 (15.0%)	3 (2.7%)	0 (0.0%)	0 (0.0%)	113 (100.0%)	113 (100.0%)		
	**10-15**	1 (1.3%)	9 (11.3%)	5 (6.3%)	10 (12.5%)	13 (16.3%)	23 (28.8%)	40 (50.0%)	34 (42.5%)	21 (26.3%)	3 (3.8%)	0 (0.0%)	1 (1.3%)	80 (100.0%)	80 (100.0%)		
	**≥16**	4 (5.8%)	15 (21.7%)	7 (10.1%)	6 (8.7%)	16 (23.2%)	9 (13.0%)	28 (40.6%)	36 (52.2%)	13 (18.8%)	0 (0.0%)	1 (1.4%)	3 (4.3%)	69 (100.0%)	69 (100.0%)	0.161	0.104
**Number of single crowns delivered per month**	**< 10**	2 (3.8%)	10 (18.9%)	5 (9.4%)	5 (9.4%)	12 (22.6%)	15 (28.3%)	23 (43.4%)	20 (37.7%)	10 (18.9%)	1 (1.9%)	1 (1.9%)	2 (3.8%)	53 (100.0%)	53 (100.0%)		
	**10-20**	5 (5.4%)	13 (14.0%)	9 (9.7%)	14 (15.1%)	31 (33.3%)	23 (24.7%)	38 (40.9%)	38 (40.9%)	10 (10.8%)	4 (4.3%)	0 (0.0%)	1 (1.1%)	93 (100.0%)	93 (100.0%)	0.007	0.365

	**> 20**	3 (2.6%)	16 (13.8%)	3 (2.6%)	9 (7.8%)	19 (16.4%)	28 (24.1%)	60 (51.7%)	61 (52.6%)	31 (26.7%)	1 (0.9%)	0 (0.0%)	1 (0.9%)	116 (100.0%)	116 (100.0%)		
**Practice Type**	**MOH**	5 (4.8%)	21 (20.0%)	4 (3.8%)	13 (12.4%)	34 (32.4%)	26 (24.8%)	44 (41.9%)	42 (40.0%)	18 (17.1%)	2 (1.9%)	0 (0.0%)	1 (1.0%)	105 (100.0%)	105 (100.0%)		
	**Private**	2 (3.7%)	6 (11.1%)	3 (5.6%)	3 (5.6%)	3 (5.6%)	8 (14.8%)	27 (50.0%)	35 (64.8%)	19 (35.2%)	2 (3.7%)	0 (0.0%)	0 (0.0%)	54 (100.0%)	54 (100.0%)		
	**Edu. Inst.**	0 (0.0%)	1 (5.0%)	3 (15.0%)	3 (15.0%)	3 (15.0%)	6 (30.0%)	10 (50.0%)	8 (40.0%)	4 (20.0%)	0 (0.0%)	0 (0.0%)	2 (10.0%)	20 (100.0%)	20 (100.0%)		
	**Multiple**	3 (3.6%)	11 (13.3%)	7 (8.4%)	9 (10.8%)	22 (26.5%)	26 (31.3%)	40 (48.2%)	34 (41.0%)	10 (12.0%)	2 (2.4%)	1 (1.2%)	1 (1.2%)	83 (100.0%)	83 (100.0%)	0.016	0.014

Table S3: Number and percentage of answers for single-unit crown materials in the subgingival margin with adequate preparation height (≥3.0 mm) for anterior and posterior teeth according to age, specialty, years of experience, number of single crowns delivered per month, and practice type.

		(PFM)		Feldspathic		Lithium disilicate		Monolithic zirconia		Layered zirconia		Other		Total		P value	
**Variable**		**Ant**	**Post**	**Ant**	**Post**	**Ant**	**Post**	**Ant**	**Post**	**Ant**	**Post**	**Ant**	**Post**	**Ant**	**Post**	**Ant**	**Post**
**Age**	**20-30**	3 (11.5%)	2 (7.7%)	5 (19.2%)	0 (0.0%)	11 (42.3%)	1 (3.8%)	0 (0.0%)	18 (69.2%)	7 (26.9%)	5 (19.2%)	0 (0.0%)	0 (0.0%)	26 (100.0%)	26 (100.0%)		
	**31-40**	9 (6.0%)	24 (15.9%)	7 (4.6%)	5 (3.3%)	84 (55.6%)	15 (9.9%)	9 (6.0%)	76 (50.3%)	41 (27.2%)	31 (20.5%)	1 (0.7%)	0 (0.0%)	151 (100.0%)	151 (100.0%)		
	**41-50**	2 (3.0%)	8 (11.9%)	3 (4.5%)	5 (7.5%)	34 (50.7%)	5 (7.5%)	1 (1.5%)	24 (35.8%)	27 (40.3%)	25 (37.3%)	0 (0.0%)	0 (0.0%)	67 (100.0%)	67 (100.0%)		
	**≥51**	0 (0.0%)	2 (11.1%)	2 (11.1%)	0 (0.0%)	7 (38.9%)	2 (11.1%)	1 (5.6%)	9 (50.0%)	6 (33.3%)	4 (22.2%)	2 (11.1%)	1 (5.6%)	18 (100.0%)	18 (100.0%)	0.003	0.01
**Specialty**	**General Dentist**	6 (6.7%)	14 (15.7%)	10 (11.2%)	0 (0.0%)	37 (41.6%)	7 (7.9%)	5 (5.6%)	43 (48.3%)	30 (33.7%)	25 (28.1%)	1 (1.1%)	0 (0.0%)	89 (100.0%)	89 (100.0%)		
	**Prosthodontist**	5 (3.5%)	12 (8.5%)	4 (2.8%)	10 (7.1%)	82 (58.2%)	11 (7.8%)	3 (2.1%)	74 (52.5%)	45 (31.9%)	33 (23.4%)	2 (1.4%)	1 (0.7%)	141 (100.0%)	141 (100.0%)		
	**Others**	3 (9.4%)	10 (31.3%)	3 (9.4%)	0 (0.0%)	17 (53.1%)	5 (15.6%)	3 (9.4%)	10 (31.3%)	6 (18.8%)	7 (21.9%)	0 (0.0%)	0 (0.0%)	32 (100.0%)	32 (100.0%)	0.103	0.006
**Experience**	**≤10**	7 (6.2%)	17 (15.0%)	10 (8.8%)	3 (2.7%)	61 (54.0%)	9 (8.0%)	4 (3.5%)	64 (56.6%)	30 (26.5%)	20 (17.7%)	1 (0.9%)	0 (0.0%)	113 (100.0%)	113 (100.0%)		
	**10-15**	6 (7.5%)	11 (13.8%)	3 (3.8%)	4 (5.0%)	45 (56.3%)	8 (10.0%)	6 (7.5%)	33 (41.3%)	20 (25.0%)	24 (30.0%)	0 (0.0%)	0 (0.0%)	80 (100.0%)	80 (100.0%)		
	**≥16**	1 (1.4%)	8 (11.6%)	4 (5.8%)	3 (4.3%)	30 (43.5%)	6 (8.7%)	1 (1.4%)	30 (43.5%)	31 (44.9%)	21 (30.4%)	2 (2.9%)	1 (1.4%)	69 (100.0%)	69 (100.0%)	0.08	0.361
**Number of single crowns delivered per month**	**< 10**	4 (7.5%)	13 (24.5%)	5 (9.4%)	0 (0.0%)	27 (50.9%)	3 (5.7%)	5 (9.4%)	29 (54.7%)	11 (20.8%)	7 (13.2%)	1 (1.9%)	1 (1.9%)	53 (100.0%)	53 (100.0%)		
	**10-20**	8 (8.6%)	15 (16.1%)	6 (6.5%)	3 (3.2%)	35 (37.6%)	8 (8.6%)	4 (4.3%)	44 (47.3%)	40 (43.0%)	23 (24.7%)	0 (0.0%)	0 (0.0%)	93 (100.0%)	93 (100.0%)		
	**> 20**	2 (1.7%)	8 (6.9%)	6 (5.2%)	7 (6.0%)	74 (63.8%)	12 (10.3%)	2 (1.7%)	54 (46.6%)	30 (25.9%)	35 (30.2%)	2 (1.7%)	0 (0.0%)	116 (100.0%)	116 (100.0%)	0.005	0.015

**Practice Type**	**MOH**	8 (7.6%)	15 (14.3%)	5 (4.8%)	2 (1.9%)	54 (51.4%)	6 (5.7%)	3 (2.9%)	56 (53.3%)	35 (33.3%)	26 (24.8%)	0 (0.0%)	0 (0.0%)	105 (100.0%)	105 (100.0%)		
	**Private**	1 (1.9%)	1 (1.9%)	4 (7.4%)	2 (3.7%)	29 (53.7%)	9 (16.7%)	1 (1.9%)	25 (46.3%)	17 (31.5%)	17 (31.5%)	2 (3.7%)	0 (0.0%)	54 (100.0%)	54 (100.0%)		
	**Edu. Inst.**	1 (5.0%)	10 (50.0%)	4 (20.0%)	0 (0.0%)	8 (40.0%)	2 (10.0%)	3 (15.0%)	7 (35.0%)	4 (20.0%)	1 (5.0%)	0 (0.0%)	0 (0.0%)	20 (100.0%)	20 (100.0%)		
	**Multiple**	4 (4.8%)	10 (12.0%)	4 (4.8%)	6 (7.2%)	45 (54.2%)	6 (7.2%)	4 (4.8%)	39 (47.0%)	25 (30.1%)	21 (25.3%)	1 (1.2%)	1 (1.2%)	83 (100.0%)	83 (100.0%)	0.185	<0.001

Table S4: Number and percentage of answers for single-unit crown luting agents in the subgingival margin with adequate preparation height (≥3.0 mm) for anterior and posterior teeth according to age, specialty, years of experience, number of single crowns delivered per month, and practice type.

		Glass ionomer		Resin-Modified Glass Ionomer		Self-adhesive cement		Dual-cure resin cement		Light cure resin cement		Other		Total		P value	
**Variable**		**Ant**	**Post**	**Ant**	**Post**	**Ant**	**Post**	**Ant**	**Post**	**Ant**	**Post**	**Ant**	**Post**	**Ant**	**Post**	**Ant**	**Post**
**Age**	**20-30**	3 (11.5%)	4 (15.4%)	5 (19.2%)	3 (11.5%)	2 (7.7%)	5 (19.2%)	11 (42.3%)	14 (53.8%)	5 (19.2%)	0 (0.0%)	0 (0.0%)	0 (0.0%)	26 (100.0%)	26 (100.0%)		
	**31-40**	24 (15.9%)	33 (21.9%)	16 (10.6%)	22 (14.6%)	36 (23.8%)	39 (25.8%)	65 (43.0%)	51 (33.8%)	9 (6.0%)	3 (2.0%)	1 (0.7%)	3 (2.0%)	151 (100.0%)	151 (100.0%)		
	**41-50**	9 (13.4%)	19 (28.4%)	9 (13.4%)	11 (16.4%)	16 (23.9%)	11 (16.4%)	27 (40.3%)	24 (35.8%)	4 (6.0%)	0 (0.0%)	2 (3.0%)	2 (3.0%)	67 (100.0%)	67 (100.0%)		
	**≥51**	1 (5.6%)	7 (38.9%)	5 (27.8%)	3 (16.7%)	2 (11.1%)	3 (16.7%)	7 (38.9%)	3 (16.7%)	2 (11.1%)	0 (0.0%)	1 (5.6%)	2 (11.1%)	18 (100.0%)	18 (100.0%)	0.024	0.274
**Specialty**	**General Dentist**	17 (19.1%)	24 (27.0%)	11 (12.4%)	13 (14.6%)	14 (15.7%)	16 (18.0%)	39 (43.8%)	34 (38.2%)	8 (9.0%)	1 (1.1%)	0 (0.0%)	1 (1.1%)	89 (100.0%)	89 (100.0%)		
	**Prosthodontist**	11 (7.8%)	28 (19.9%)	22 (15.6%)	23 (16.3%)	37 (26.2%)	38 (27.0%)	57 (40.4%)	47 (33.3%)	10 (7.1%)	0 (0.0%)	4 (2.8%)	5 (3.5%)	141 (100.0%)	141 (100.0%)		
	**Others**	9 (28.1%)	11 (34.4%)	2 (6.3%)	3 (9.4%)	5 (15.6%)	4 (12.5%)	14 (43.8%)	11 (34.4%)	2 (6.3%)	2 (6.3%)	0 (0.0%)	1 (3.1%)	32 (100.0%)	32 (100.0%)	0.085	0.051
**Experience**	**≤10**	11 (9.7%)	23 (20.4%)	13 (11.5%)	17 (15.0%)	27 (23.9%)	25 (22.1%)	48 (42.5%)	44 (38.9%)	11 (9.7%)	1 (0.9%)	3 (2.7%)	3 (2.7%)	113 (100.0%)	113 (100.0%)		
	**10-15**	13 (16.3%)	16 (20.0%)	9 (11.3%)	9 (11.3%)	18 (22.5%)	25 (31.3%)	34 (42.5%)	26 (32.5%)	6 (7.5%)	2 (2.5%)	0 (0.0%)	2 (2.5%)	80 (100.0%)	80 (100.0%)		
	**≥16**	13 (18.8%)	24 (34.8%)	13 (18.8%)	13 (18.8%)	11 (15.9%)	8 (11.6%)	28 (40.6%)	22 (31.9%)	3 (4.3%)	0 (0.0%)	1 (1.4%)	2 (2.9%)	69 (100.0%)	69 (100.0%)	0.255	0.163
**Number of single crowns delivered per month**	**< 10**	6 (11.3%)	13 (24.5%)	5 (9.4%)	8 (15.1%)	13 (24.5%)	10 (18.9%)	25 (47.2%)	19 (35.8%)	3 (5.7%)	1 (1.9%)	1 (1.9%)	2 (3.8%)	53 (100.0%)	53 (100.0%)		
	**10-20**	17 (18.3%)	22 (23.7%)	16 (17.2%)	16 (17.2%)	24 (25.8%)	21 (22.6%)	30 (32.3%)	31 (33.3%)	4 (4.3%)	1 (1.1%)	2 (2.2%)	2 (2.2%)	93 (100.0%)	93 (100.0%)		
	**> 20**	14 (12.1%)	28 (24.1%)	14 (12.1%)	15 (12.9%)	19 (16.4%)	27 (23.3%)	55 (47.4%)	42 (36.2%)	13 (11.2%)	1 (0.9%)	1 (0.9%)	3 (2.6%)	116 (100.0%)	116 (100.0%)	0.107	0.995

**Practice Type**	**MOH**	14 (13.3%)	27 (25.7%)	15 (14.3%)	18 (17.1%)	23 (21.9%)	21 (20.0%)	40 (38.1%)	36 (34.3%)	10 (9.5%)	0 (0.0%)	3 (2.9%)	3 (2.9%)	105 (100.0%)	105 (100.0%)		
	**Private**	13 (24.1%)	13 (24.1%)	7 (13.0%)	6 (11.1%)	4 (7.4%)	9 (16.7%)	25 (46.3%)	23 (42.6%)	5 (9.3%)	2 (3.7%)	0 (0.0%)	1 (1.9%)	54 (100.0%)	54 (100.0%)		
	**Edu. Inst.**	1 (5.0%)	4 (20.0%)	3 (15.0%)	5 (25.0%)	5 (25.0%)	4 (20.0%)	10 (50.0%)	6 (30.0%)	1 (5.0%)	0 (0.0%)	0 (0.0%)	1 (5.0%)	20 (100.0%)	20 (100.0%)		
	**Multiple**	9 (10.8%)	19 (22.9%)	10 (12.0%)	10 (12.0%)	24 (28.9%)	24 (28.9%)	35 (42.2%)	27 (32.5%)	4 (4.8%)	1 (1.2%)	1 (1.2%)	2 (2.4%)	83 (100.0%)	83 (100.0%)	0.214	0.581

Table S5: Number and percentage of answers for single-unit crown materials in short preparations (<3.0 mm) with supragingival or equigingival margins for anterior and posterior teeth according to age, specialty, years of experience, number of single crowns delivered per month, and practice type.

		(PFM)		Feldspathic		Lithium disilicate		Monolithic zirconia		Layered zirconia		Other		Total		P value	
**Variable**		**Ant**	**Post**	**Ant**	**Post**	**Ant**	**Post**	**Ant**	**Post**	**Ant**	**Post**	**Ant**	**Post**	**Ant**	**Post**	**Ant**	**Post**
**Age**	**20-30**	0 (0.0%)	2 (7.7%)	4 (15.4%)	0 (0.0%)	12 (46.2%)	7 (26.9%)	2 (7.7%)	12 (46.2%)	8 (30.8%)	4 (15.4%)	0 (0.0%)	1 (3.8%)	26 (100.0%)	26 (100.0%)		
	**31-40**	6 (4.0%)	16 (10.6%)	11 (7.3%)	7 (4.6%)	97 (64.2%)	43 (28.5%)	4 (2.6%)	45 (29.8%)	31 (20.5%)	34 (22.5%)	2 (1.3%)	6 (4.0%)	151 (100.0%)	151 (100.0%)		
	**41-50**	3 (4.5%)	6 (9.0%)	5 (7.5%)	6 (9.0%)	39 (58.2%)	12 (17.9%)	0 (0.0%)	25 (37.3%)	18 (26.9%)	16 (23.9%)	2 (3.0%)	2 (3.0%)	67 (100.0%)	67 (100.0%)		
	**≥51**	1 (5.6%)	3 (16.7%)	0 (0.0%)	0 (0.0%)	8 (44.4%)	2 (11.1%)	4 (22.2%)	9 (50.0%)	4 (22.2%)	1 (5.6%)	1 (5.6%)	3 (16.7%)	18 (100.0%)	18 (100.0%)	0.01	0.029
**Specialty**	**General Dentist**	2 (2.2%)	10 (11.2%)	16 (18.0%)	1 (1.1%)	44 (49.4%)	23 (25.8%)	4 (4.5%)	27 (30.3%)	22 (24.7%)	27 (30.3%)	1 (1.1%)	1 (1.1%)	89 (100.0%)	89 (100.0%)		
	**Prosthodontist**	6 (4.3%)	16 (11.3%)	4 (2.8%)	11 (7.8%)	87 (61.7%)	25 (17.7%)	5 (3.5%)	58 (41.1%)	35 (24.8%)	22 (15.6%)	4 (2.8%)	9 (6.4%)	141 (100.0%)	141 (100.0%)		
	**Others**	2 (6.3%)	1 (3.1%)	0 (0.0%)	1 (3.1%)	25 (78.1%)	16 (50.0%)	1 (3.1%)	6 (18.8%)	4 (12.5%)	6 (18.8%)	0 (0.0%)	2 (6.3%)	32 (100.0%)	32 (100.0%)	0.002	0.001
**Experience**	**≤10**	4 (3.5%)	14 (12.4%)	7 (6.2%)	4 (3.5%)	67 (59.3%)	31 (27.4%)	3 (2.7%)	39 (34.5%)	30 (26.5%)	21 (18.6%)	2 (1.8%)	4 (3.5%)	113 (100.0%)	113 (100.0%)		
	**10-15**	3 (3.8%)	7 (8.8%)	10 (12.5%)	4 (5.0%)	47 (58.8%)	19 (23.8%)	3 (3.8%)	25 (31.3%)	15 (18.8%)	21 (26.3%)	2 (2.5%)	4 (5.0%)	80 (100.0%)	80 (100.0%)		
	**≥16**	3 (4.3%)	6 (8.7%)	3 (4.3%)	5 (7.2%)	42 (60.9%)	14 (20.3%)	4 (5.8%)	27 (39.1%)	16 (23.2%)	13 (18.8%)	1 (1.4%)	4 (5.8%)	69 (100.0%)	69 (100.0%)	0.777	0.719
**Number of single crowns delivered per month**	**< 10**	3 (5.7%)	5 (9.4%)	4 (7.5%)	1 (1.9%)	30 (56.6%)	17 (32.1%)	5 (9.4%)	14 (26.4%)	10 (18.9%)	13 (24.5%)	1 (1.9%)	3 (5.7%)	53 (100.0%)	53 (100.0%)		
	**10-20**	5 (5.4%)	11 (11.8%)	5 (5.4%)	2 (2.2%)	50 (53.8%)	18 (19.4%)	1 (1.1%)	42 (45.2%)	32 (34.4%)	16 (17.2%)	0 (0.0%)	4 (4.3%)	93 (100.0%)	93 (100.0%)		
	**> 20**	2 (1.7%)	11 (9.5%)	11 (9.5%)	10 (8.6%)	76 (65.5%)	29 (25.0%)	4 (3.4%)	35 (30.2%)	19 (16.4%)	26 (22.4%)	4 (3.4%)	5 (4.3%)	116 (100.0%)	116 (100.0%)	0.015	0.216

**Practice Type**	**MOH**	4 (3.8%)	10 (9.5%)	2 (1.9%)	2 (1.9%)	68 (64.8%)	22 (21.0%)	5 (4.8%)	46 (43.8%)	24 (22.9%)	18 (17.1%)	2 (1.9%)	7 (6.7%)	105 (100.0%)	105 (100.0%)		
	**Private**	2 (3.7%)	2 (3.7%)	13 (24.1%)	3 (5.6%)	33 (61.1%)	16 (29.6%)	1 (1.9%)	15 (27.8%)	4 (7.4%)	17 (31.5%)	1 (1.9%)	1 (1.9%)	54 (100.0%)	54 (100.0%)		
	**Edu. Inst.**	1 (5.0%)	4 (20.0%)	1 (5.0%)	1 (5.0%)	9 (45.0%)	6 (30.0%)	3 (15.0%)	6 (30.0%)	6 (30.0%)	3 (15.0%)	0 (0.0%)	0 (0.0%)	20 (100.0%)	20 (100.0%)		
	**Multiple**	3 (3.6%)	11 (13.3%)	4 (4.8%)	7 (8.4%)	46 (55.4%)	20 (24.1%)	1 (1.2%)	24 (28.9%)	27 (32.5%)	17 (20.5%)	2 (2.4%)	4 (4.8%)	83 (100.0%)	83 (100.0%)	<0.001	0.25

Table S6: Number and percentage of answers for single-unit crown luting agents in short preparations (<3.0 mm) with supragingival or equigingival margins for anterior and posterior teeth according to age, specialty, years of experience, number of single crowns delivered per month, and practice type.

		Glass ionomer		Resin-Modified Glass Ionomer		Self-adhesive cement		Dual-cure resin cement		Light cure resin cement		Other		Total		P value	
**Variable**		**Ant**	**Post**	**Ant**	**Post**	**Ant**	**Post**	**Ant**	**Post**	**Ant**	**Post**	**Ant**	**Post**	**Ant**	**Post**	**Ant**	**Post**
**Age**	**20-30**	1 (3.8%)	1 (3.8%)	2 (7.7%)	1 (3.8%)	4 (15.4%)	7 (26.9%)	14 (53.8%)	16 (61.5%)	4 (15.4%)	0 (0.0%)	1 (3.8%)	1 (3.8%)	26 (100.0%)	26 (100.0%)		
	**31-40**	5 (3.3%)	11 (7.3%)	4 (2.6%)	12 (7.9%)	31 (20.5%)	36 (23.8%)	80 (53.0%)	79 (52.3%)	30 (19.9%)	10 (6.6%)	1 (0.7%)	3 (2.0%)	151 (100.0%)	151 (100.0%)		
	**41-50**	3 (4.5%)	13 (19.4%)	5 (7.5%)	3 (4.5%)	20 (29.9%)	14 (20.9%)	30 (44.8%)	33 (49.3%)	8 (11.9%)	3 (4.5%)	1 (1.5%)	1 (1.5%)	67 (100.0%)	67 (100.0%)		
	**≥51**	1 (5.6%)	1 (5.6%)	4 (5.6%)	4 (22.2%)	3 (5.6%)	7 (38.9%)	7 (5.6%)	5 (27.8%)	2 (5.6%)	0 (0.0%)	1 (5.6%)	1 (5.6%)	18 (5.6%)	18 (100.0%)	0.047	0.045
**Specialty**	**General Dentist**	1 (1.1%)	8 (9.0%)	3 (3.4%)	5 (5.6%)	16 (18.0%)	20 (22.5%)	46 (51.7%)	47 (52.8%)	21 (23.6%)	7 (7.9%)	2 (2.2%)	2 (2.2%)	89 (100.0%)	89 (100.0%)		
	**Prosthodontist**	7 (5.0%)	15 (10.6%)	12 (8.5%)	13 (9.2%)	32 (22.7%)	37 (26.2%)	70 (49.6%)	69 (48.9%)	18 (12.8%)	3 (2.1%)	2 (1.4%)	4 (2.8%)	141 (100.0%)	141 (100.0%)		
	**Others**	2 (6.3%)	3 (9.4%)	0 (0.0%)	2 (6.3%)	10 (31.3%)	7 (21.9%)	15 (46.9%)	17 (53.1%)	5 (15.6%)	3 (9.4%)	0 (0.0%)	0 (0.0%)	32 (100.0%)	32 (100.0%)	0.095	0.689
**Experience**	**≤10**	4 (3.5%)	12 (10.6%)	4 (3.5%)	11 (9.7%)	29 (25.7%)	24 (21.2%)	56 (49.6%)	61 (54.0%)	18 (15.9%)	3 (2.7%)	2 (1.8%)	2 (1.8%)	113 (100.0%)	113 (100.0%)		
	**10-15**	3 (3.8%)	4 (5.0%)	5 (6.3%)	4 (5.0%)	12 (15.0%)	26 (32.5%)	45 (56.3%)	38 (47.5%)	14 (17.5%)	5 (6.3%)	1 (1.3%)	3 (3.8%)	80 (100.0%)	80 (100.0%)		
	**≥16**	3 (4.3%)	10 (14.5%)	6 (8.7%)	5 (7.2%)	17 (24.6%)	14 (20.3%)	30 (43.5%)	34 (49.3%)	12 (17.4%)	5 (7.2%)	1 (1.4%)	1 (1.4%)	69 (100.0%)	69 (100.0%)	0.592	0.34
**Number of single crowns delivered per month**	**< 10**	1 (1.9%)	5 (9.4%)	2 (3.8%)	4 (7.5%)	14 (26.4%)	14 (26.4%)	25 (47.2%)	23 (43.4%)	8 (15.1%)	4 (7.5%)	3 (5.7%)	3 (5.7%)	53 (100.0%)	53 (100.0%)		
	**10-20**	7 (7.5%)	17 (18.3%)	7 (7.5%)	6 (6.5%)	24 (25.8%)	24 (25.8%)	45 (48.4%)	43 (46.2%)	10 (10.8%)	3 (3.2%)	0 (0.0%)	0 (0.0%)	93 (100.0%)	93 (100.0%)		
	**> 20**	2 (1.7%)	4 (3.4%)	6 (5.2%)	10 (8.6%)	20 (17.2%)	26 (22.4%)	61 (52.6%)	67 (57.8%)	26 (22.4%)	6 (5.2%)	1 (0.9%)	3 (2.6%)	116 (100.0%)	116 (100.0%)	0.033	0.066

**Practice Type**	**MOH**	3 (2.9%)	14 (13.3%)	8 (7.6%)	11 (10.5%)	28 (26.7%)	22 (21.0%)	51 (48.6%)	53 (50.5%)	14 (13.3%)	2 (1.9%)	1 (1.0%)	3 (2.9%)	105 (100.0%)	105 (100.0%)		
	**Private**	2 (3.7%)	2 (3.7%)	2 (3.7%)	1 (1.9%)	7 (13.0%)	13 (24.1%)	26 (48.1%)	31 (57.4%)	17 (31.5%)	7 (13.0%)	0 (0.0%)	0 (0.0%)	54 (100.0%)	54 (100.0%)		
	**Edu. Inst.**	0 (0.0%)	4 (20.0%)	2 (10.0%)	2 (10.0%)	6 (30.0%)	6 (30.0%)	7 (35.0%)	6 (30.0%)	5 (25.0%)	2 (10.0%)	0 (0.0%)	0 (0.0%)	20 (100.0%)	20 (100.0%)		
	**Multiple**	5 (6.0%)	6 (7.2%)	3 (3.6%)	6 (7.2%)	17 (20.5%)	23 (27.7%)	47 (56.6%)	43 (51.8%)	8 (9.6%)	2 (2.4%)	3 (3.6%)	3 (3.6%)	83 (100.0%)	83 (100.0%)	0.101	0.07
